# Prevalence and Socio-Demographic, Academic, Health and Lifestyle Predictors of Illicit Drug/s Use among University Undergraduate Students in Finland

**DOI:** 10.3390/ijerph17145094

**Published:** 2020-07-15

**Authors:** Walid El Ansari, Abdul Salam, Sakari Suominen

**Affiliations:** 1 Department of Surgery, Hamad General Hospital, Hamad Medical Corporation, Doha 3050, Qatar; 2 College of Medicine, Qatar University, Doha 3050, Qatar; 3 School of Health and Education, University of Skovde, 541 28 Skövde, Sweden; sakari.suominen@his.se; 4 Department of Epidemiology and Biostatistics, King Fahad Specialist Hospital, Dammam 31444, Saudi Arabia; abdul.or.salam@gmail.com

**Keywords:** university students, illicit drug/s use, mental health, academic performance, sociodemographic and educational characteristics

## Abstract

Illicit drug/s use (IDU) among university students is a public health concern. We assessed the associations between socio-demographic, academic, and health and lifestyle characteristics (independent variables) and regular, occasional or never IDU (dependent variables). Data were collected across seven faculties (1177 students) at the University of Turku (Finland) via an online questionnaire. About 1.5% of the sample had regular IDU, 19% occasional IDU, and 79% never IDU. Independent predictors of ever (lifetime) IDU included males [adjusted odds ratio (AOR) 1.82, P = 0.001], not living with parents (AOR 2.59, P < 0.001), singles (AOR 0.51, P < 0.001), lower religiosity (AOR 1.49, P = 0.022), better self-rated general health (AOR 0.41, P = 0.003), higher health awareness (AOR 1.93, P = 0.014), more depressive symptoms (AOR 1.82, P = 0.004), daily smokers (AOR 3.69, P < 0.001), heavy episodic drinking (AOR 2.38, P < 0.001) and possible alcohol dependency (AOR 2.55, P < 0.001). We observed no independent associations between ever IDU with age, study discipline, perceived stress or academic performance. The 20.5% ever IDU is concerning. The compelling independent predictors of ever IDU included not living with parents, lower religiosity, daily smokers, heavy episodic drinking and possible alcohol dependency (AOR range 2.38–3.69). Education and prevention need to emphasize the negative consequences to reinforce abstinence from IDU. Health promotion could focus on beliefs and expectations about IDU and target students at risk for successful efforts.

## 1. Introduction

Early adulthood is an important time period where habits and behaviors such as substance use or illicit drug/s use (IDU) are often initiated and established [1]. The university years symbolize a time of independence and separation from parental supervision, representing occasions to sample psychoactive substances (e.g., tobacco, alcohol, illicit drug/s), and are a period in which IDU frequently increases [2].

For instance, in Colorado (USA), about 75% of college students reported lifetime use of marijuana, 65% used marijuana within the last year, 29% had a positive urine screen, and 7% of participants used it daily [3]. In the United Kingdom, 33.1% of students reported IDU [4]. Research found that roughly 25% of college students had used marijuana within the last month (5% used it daily) [5,6], and among first-year students (17–20 years old), 9.4% had cannabis use disorders [7]. In South Africa, 22% of second year and 24.1% of third year students reported cannabis use, where in the second year group, 2.7% used magic mushroom, 1.8% cocaine, 1.8% ecstasy and 0.9% used methamphetamine [8]. In the USA, about 4% of full-time college students used cocaine in the past year, and 1.4% used it in the past month [5]; among Ethiopian university students, psychoactive substances use was fairly prevalent [9]; and at 11 universities in the USA, the average of past month marijuana use was 26.2% [7]. The annual prevalence of 3,4-methylenedioxymethamphetamine (MDMA, aka ecstasy) use among college students more than doubled from 2004 to 2016 [5]. Indeed, the initiation of or IDU amongst university students is a concern worldwide [4,10,11,12,13,14,15].

Marijuana use peaks between 18–25 years of age [16], the age of most college students in most countries. This is despite that IDU exhibits wide ranging deleterious effects, including altered sensory perception, changes in mood and sense of time, impaired body movement or memory, and challenges with problem solving and thinking [17]. Students who had used cannabis ≥5 times over the past year had various cannabis-related problems [7]. IDU can result in short-term changes in perception, mood, consciousness and behavior [18] [WHO 2004], along with longer-term impaired learning and memory function [8]. Unsurprisingly, within the college milieu, there is evidence of relationships between marijuana use frequency/misuse and college degree attainment, discontinuous college enrollment, missing classes, time to graduation, academic achievement (e.g., GPA), time spent studying, and academic self-efficacy [19,20,21,22,23,24]. Particularly among young adults, IDU can lead to injuries, suicide and ultimately death [21,25,26], and emergency room visits as a consequence of MDMA use in the age group of college students continue to increase [27].

A range of variables is important when examining IDU among university populations. Hence in order to further identify predictive factors for substance use, we included several variables. These comprised gender and age [28], type of accommodation [10,11], discipline of study at university [29,30,31,32], and year of study [30,33]. Other variables included religiosity [10,11,28], depressive symptoms [4], psychological [14,15] and academic stress [34], smoking [35,36], alcohol consumption [11,30,37], and financial burdens [36] among others. The rationale for including these sociodemographic, academic, and health and lifestyle variables is that previous published studies have shown that these variables are associated with IDU among university students. Hence including and controlling for such variables is important when assessing the correlates of IDU and the associations between such use and a wide range of characteristics across university students.

The literature suggests knowledge gaps. Whilst some recent studies exist concerning alcohol consumption among university students in Finland [38], the very few studies that examined IDU among Finnish university students are quite outdated. For instance, in the 90s, 3.6% of female and 3.1% of male students reported the mixed use of psychiatric drugs and alcohol with the aim of getting high, at least once [39]. In the 80s, research among Finnish university, nursing, and drama students reported that 5% of students had taken illicit drugs, and among the drama students, the rate was 38% [40]. This is despite that a 2010 survey of the general population showed that almost every sixth person in Finland aged 15–64 years had experimented with drugs at least once in their lifetime and 17.9% of drug-related deaths in 2010 were individuals < 25 years of age [41]. Although alcohol is the foremost substance of abuse in Finland, mounting concerns has led to monitoring of IDU-related health consequences, including mortality [42]. A recent report of patterns and trends in mortality among illicit drug users in Finland found that predominant primary drug by gender and by age-group included opiates among males and stimulants among females [43]. 

The current study bridges these knowledge gaps to survey a sample of students in Finland, utilizing a variety of sociodemographic, academic, and health and lifestyle variables, to describe lifetime prevalence of IDU, compare the bivariate relationships of IDU with these variables, and assess the independent predictors associated with IDU (multiple logistic regression analysis). These aspects highlight the importance of the study, and to the best of our knowledge, this study could be the first to incorporate and mobilize many variables in order to grasp the broader representation of IDU across undergraduates in Finland. The specific objectives were to:

- Illustrate selected socio-demographic, academic, and health and lifestyle characteristics of the sample and the lifetime prevalence of IDU;
- Appraise a variety of variables by IDU status (regular, occasional, never) (bivariate analysis); and,
- Assess and compare the variables associated with ever (lifetime) IDU (i.e. regular and occasional together) controlling for all other variables (multiple logistic regression analysis).

## 2. Materials and Methods 

### 2.1. Ethics, Sample, and Data Collection

Initial ‘invitation to participate’ e-mails were sent to all undergraduates at all faculties at the University outlining the goals and objectives of the research and motivating students to go online and complete the survey. Participation was voluntary and anonymous (no academic or monetary incentives were provided), and data were confidential and protected at all times. Students were informed that by completing the online survey, they consent to partake in the study. Fourteen days after the initial email invitation of students, a follow up reminder email was sent again to all undergraduates. In addition, three posters about the study were exhibited at the students’ cafeteria at the University, and a reminder was announced on the University intraweb. A pilot survey was undertaken first (May 2013, random sample, 200 students) stratified by faculties. Very few participants reported any comprehensibility challenges related to the English questionnaire, and the amount of missing values related to items that reasonably could be expected to be answered by all students was negligible. The main survey was then launched utilizing the unmodified questionnaire (September 2013). The pilot sample was excluded from the final eligible sample that included 4387 undergraduates at the University of Turku, Finland. The University Research and Ethics Committee authorized the study (Approval # Lausunto 10/2010), and data were collected using a secure online self-administered English questionnaire (2013–2014).

As students completed the online survey and ‘submitted’ their completed questionnaires, their responses were saved and directed to the Student Management Office at the University. This Office gathered the online responses, and data were electronically entered into an excel sheet ensuring high quality assurance. After completion of this phase, the data was sent to the research team who electronically imported the data (no identifiers) into the Statistical Package for Social Sciences Version 24 (SPSS 24, IBM Corp., Armonk, NY, USA) for analysis. The total number of responses received was 1177. Students’ mean age was about ≈23 (SD 5) years and 832 (70.4%) were females. Based on the number of returned questionnaires, the response rate was about 27%.

### 2.2. Health and Wellbeing Questionnaire 

The self-administered questionnaire gathered general health data: socio-demographic (sex, age, year of study, living arrangements during university terms); health (self-rated general health, health awareness); lifestyle (IDU, smoking, heavy episodic drinking, problem drinking, possible alcohol dependence); mental wellbeing variables (depressive symptoms, perceived stress), university related educational questions (academic achievement compared to peers), and information on religiosity and financial burdens. The tool was used and field-tested across many student populations [44,45,46,47,48,49,50,51,52,53,54]. Variables with several response options were later dichotomized as shown in Table 1. 

#### 2.2.1. Sociodemographic Variables

Age, sex and year of study at university were based on self-reports. Age was used as a continuous variable.

Accommodation (living arrangements) during semester time: “Where do you live during university/college term time?”, dichotomized into ‘living with parents’ vs. ‘not living with parents’ [50].

Marital status: What is your marital status? Response options included single, married, or other (please specify), dichotomized into ’single’ vs. ’married or in relationship’ [50].

Discipline of study: students were asked about the faculty they were enrolled at in the University of Turku, and discipline they were studying. For the current analysis we collapsed the seven faculties into five.

Financial burden/s: “To what extent do you feel burdened in the following areas?” “Financial situation”, 1 = ‘not at all’, 6 = ‘Very strongly’, later dichotomized into 1, 2, 3, 4 = 1 vs. 5, 6 = 2 (strongly/very strongly) [55]. 

Religiosity (personal importance of religious faith): the extent to which participants agreed/disagreed with the statement: “My religion is very important for my life”, 1 = ‘strongly agree’, 2 = ‘somewhat agree’, 3 = ‘neither agree nor disagree’, 4 = ‘somewhat disagree’, and 5 = ‘strongly disagree’, later recoded into two categories based on agreement/disagreement (1, 2, 3 = 1 vs. 4, 5 = 2) [50]. 

#### 2.2.2. Academic Achievement 

The current study conceptualized and measured academic performance using students’ subjective comparative appraisal of their overall performance in comparison with their peers [53]: “How do you rate your performance in comparison with your fellow students?” 1 = ‘much better’, 2 = ‘better’, 3 = ‘same’, 4 = ‘worse’, 5=‘much worse’, later dichotomized based on perceived better performance (3, 4, 5 = 1 vs. 1, 2 = 2).

#### 2.2.3. Health Variables 

Self-rated general health: “How would you describe your general health?” (1 = ‘poor’, 5 = ‘excellent’) (adopted from [56]). 

Health awareness: “To what extent do you keep an eye on your health?” (1 = ‘not at all’, 4 = ‘very much’) [57]. 

Depressive symptoms (20 items): using the Modified Beck Depression Inventory (M-BDI) [58,59]. Sample items included: “I feel sad,” “I feel I am being punished,” “I have thoughts of killing myself,” “I have lost interest in other people,” “I have to force myself to do anything,” “I am worried about my appearance,” and “I have no appetite”. BDI computes a single score for individual respondents by summing their responses for all items of the scale. We used the 5th quintile to categorize depressive symptoms as high. 

Perceived Stress Scale (4 Items): Cohen’s Perceived Stress Scale (PSS) in its four item short form [60] assessed the extent to which participants considered life situations to be stressful. PSS-4 is a simple psychological instrument that measures the degree to which situations in one’s life over the past month are appraised as stressful. The questions are general items designed to detect how unpredictable, uncontrollable, and overloaded respondents find their lives. All items began with: “In the past month, how often have you felt...?” (5 point scale: 0 = ‘never’, 1 = ‘almost never’, 2 = ‘sometimes’, 3 = ‘fairly often’, 4 = ‘very often’). In our sample, Cronbach’s alpha of PSS was 0.75. A median split (median = 12) categorized the variable into ‘Higher’ and ‘Lower’ stress (higher scores = more perceived stress). 

#### 2.2.4. Lifestyle Variables 

Illicit drug/s use: “Have you ever use/used drugs?” (‘Yes, regularly’; ‘Yes, but only a few times’; ‘Never’) [28]. 

Smoking: “Within the last three months, how often did you smoke? (cigarettes, pipe, cigarillos, cigars)” (daily, occasionally, never) [61]. 

Heavy episodic drinking (frequency): “Think back over the last two weeks. How many times (if any) have you had ≥ 5 alcoholic drinks at a sitting?” [A “drink” is a glass/bottle/can of beer (≈50 cL), a glass/bottle/can of cider (≈50 cL), 2 glasses/bottles of alcopops (≈50 cL), a glass of wine (≈15 cL), a glass of spirits (≈5 cL) or a mixed drink] [62]. Responses were dichotomized into no heavy episodic drinking (<1 time) vs. heavy episodic drinking (≥1 time). 

Problem drinking (four items): An alcoholism-screening CAGE test [63] comprising four questions (Have you ever felt you should cut down on your drinking? Have people annoyed you by criticizing your drinking? Have you ever-felt bad or guilty about your drinking? Have you ever had a drink in the morning to get rid of a hangover? (Eye opener). Each question is answered either “yes” or “no.” Two or more affirmative answers suggested problem drinking. We categorized respondents as non-problem (<2 positive responses) vs. problem drinkers (≥2 positive responses). 

Possible alcohol dependence (four items): ≥ 3 positive CAGE responses [63] can suggest alcohol dependence. We categorized respondents as not possible alcohol dependence (<3 positive responses) vs. possible alcohol dependence (≥ 3 positive responses). 

### 2.3. Statistical Analysis

Descriptive and inferential statistics were used to characterize the study sample and test hypotheses. To assess the different status of IDU, we stratified the dependent variable “have you ever use/used drug/s?” into three; ‘regular user’, ‘occasional user’ (only a few times) and ‘never user’. Binomial distribution measured the prevalence of IDU among university students at Finland. Descriptive results for all quantitative variables (e.g., age) are presented as mean ± standard deviation (SD; for normally distributed data), while numbers (percentage) were reported for all qualitative variables (e.g., gender) for the whole sample and for each IDU status. Using bivariate analysis, the prevalence, odds ratio (OR) and 95% confidence interval (CI) for socio demographic, academic performance compared to peers, and lifestyle variables (alcohol consumption behaviors) were calculated separately for each IDU status (regular vs. never users; occasional vs. never users; and never vs. ever users).

Multiple binary logistic regression models were used to identify significant independent factors associated with IDU (regular or occasional user vs. never users). Several of the continuous variables were dichotomized (e.g., BDI score for depressive symptoms, perceived stress) in order to interpret the results of the log regression models better. The OR for each IDU status was adjusted for; sex, age, discipline of study at University, accommodation during-semester, marital status, religiosity status, financial burden/s, self-rated general health, health awareness, depressive symptoms, perceived stress, smoking, heavy episodic drinking in the last 2 weeks, CAGE score, and academic performance compared to one’s peers. Wald test computed on each factor determined which were significant. Adjusted Odds ratio and 95% confidence interval for the adjusted odds ratio were reported. A “P” value <0.05 (two-tailed) was considered statistically significant. The Hosmer-Lemeshow Goodness-of-fit statistics were used to determine whether the model adequately describes the data. All statistical analyses were performed using SPSS Version 24.

## 3. Results

### 3.1. Participating Faculties and Disciplines

Participating students were enrolled in all seven faculties of the University of Turku (Humanities, Mathematics and Natural Sciences, Medicine, Law, Social Sciences, Education, and Economics). Many disciplines of study were included in the study e.g., adult education, special education, pedagogy, languages, philosophy, law, accounting, finance, economics, marketing, medicine, nursing, dentistry, psychology, biomedicine, bioscience, biochemistry, biology, chemistry, mathematics, geography, history, political or social science, computer science, information technology, and biotechnology.

### 3.2. Sample Characteristics 

The sample comprised 70.4% females and 29.6% males, and mean age was 23 ± 5.2 years (Table 1). About half the respondents (47.2%) were 1st year students, and another third (29.4%) were 2nd years. Slightly more than half the sample attended Technology and Science or Humanities disciplines. More students did not live with their parents (66.3%), and half the sample were single. 

About 1.5% of the students reported regular IDU, 19% occasional IDU, and 79% never IDU. More than half the respondents (60.2%) ’strongly or somewhat disagreed’ that religion was important in their life. The majority reported that finances was not a burden (83%); 92.6% felt their health was ’good, very good or excellent’; and the majority (86.4%) had high health awareness. About one fifth of respondents had high depressive symptoms (≥ 5th quintile), and 60.6% had high (≥ median) perceived stress. 

In terms of lifestyle, most students were non- or occasional smokers; 66.2% reported ≥ 1 heavy episodic drinking session during the last 2 weeks; and, 28.8% and 8.7% reported possible problem drinking or possible alcohol dependence respectively. About 1.5% of the sample regular IDU, 19.6% (17.4% females, 24% males) had occasional use (a few times), and 79% (81.8% females, 73% males) never used illicit drug/s. Academically, most respondents (84.6%) felt their academic performance compared to peers was the ’same, better or much better’.

About 30% of the respondents indicated the type/s of illicit drug/s that they had used (data not presented). The most common IDU reported were marijuana and cannabis; other illicit drug/s used included amphetamines, cocaine, opium, ketamine, ephedrine, ecstasy, hallucinogenic mushrooms, LSD, psilocybin, dextromethorphan, codeine, "modified drugs", subutex (opioid used to treat opioid use disorder), benzodiazepine, GHB (Gamma Hydroxybutyrate, a central nervous system depressant), ’nitros’, and designer drugs (data not presented).

### 3.3. Variables Associated with Regular Illicit Drug/s Use (Bivariate Analyses) 

Employing bivariate analyses, Table 2 depicts that regular IDU was significantly more likely among males, those with poor/fair self-rated general health, or daily smokers. In addition, regular IDU was significantly more likely among those reporting higher depressive symptoms, and possible problem drinking or alcohol dependence. 

### 3.4. Variables Associated with Occasional Illicit Drug/s Use (Bivariate Analyses) 

Occasional IDU was positively significantly associated with males, financial burdens, lower religiosity, daily smokers, and those reporting higher depressive symptoms, heavy episodic drinking, possible problem drinking or possible alcohol dependence. Occasional IDU was negatively significantly associated with being single, and among students with ’good/very good/excellent’ self-rated general health. Studying Technology and Science at the university was associated with lower odds of occasional IDU (Table 2).

### 3.5. Variables Associated with Never Illicit Drug/s Use (Bivariate Analyses) 

Never IDU was significantly negatively associated with most health damaging variables. Never IDU was less likely among males, younger age, not living with parents, those having financial burdens, lower religiosity, daily smokers, those with higher depressive symptoms or higher perceived stress, and those reporting alcohol misuse (heavy episodic drinking, possible problem drinking or possible alcohol dependence). 

Never IDU was significantly more likely among those with good self-rated general health. Studying Technology and Science at the university was associated with higher odds of never IDU (Table 2).

### 3.6. Independent Predictors of Ever (lifetime) Illicit Drug/s Use (Multiple Logistic Regression) 

Independent positive predictors of ever (lifetime) IDU included being male, not living with parents, lower religiosity, higher health awareness, daily smoking, higher depressive symptoms, heavy episodic drinking, and possible alcohol dependence. Being single, ’good/very good/excellent’ self-rated general health negatively predicted ever (lifetime) IDU (Table 3).

## 4. Discussion

IDU among college students is a global public health concern. To the best of our knowledge, this study could be the first to include a wide range of socio-demographic, academic, and health and lifestyle characteristics in order to assess the broader picture of IDU across undergraduates in Finland. Employing anonymously collected data from a sizeable sample across a wide variety of academic/scientific disciplines, the current study described the lifetime prevalence of IDU, and analyzed the variables associated with regular, occasional, and never IDU among university students. 

In the current study, a total of 22.1% reported a lifetime prevalence of any illicit drug/s (ever IDU), comparable to the 2016 National Survey in the USA, where 20% of full-time college students reported using marijuana in the past month [64]. Similarly, across 8182 undergraduates at 8 Canadian post-secondary institutions, 17.5% used marijuana and 3.5% used other illicit drugs over the past month [65], which add to 21%, a rate comparable to ours. However, our observed ever IDU was less than the 30.2% ever IDU level reported across universities in England, Wales, and Northern Ireland, using the same questionnaire [28]; less than that of universities in Switzerland (44% lifetime IDU) [14]; and less that of a representative sample in the USA, where 37% of college students reported ever IDU [66]. 

As for regular IDU, our observed 1.5% rate was considerably less the 4.8% level found across students at seven universities in the United Kingdom, using the same questionnaire [28]. Across the current sample of students, the most common IDU reported were marijuana and cannabis, followed by a variety of other illicit drug/s, in line with others where the highest weekly consumption was for cannabis 26.5% (3.1% daily), followed by tranquillizers, cocaine, hallucinogens, ecstasy, and amphetamines [67]. 

Despite the fact that the 22.1% ever IDU observed among the current sample of Finnish students seems less than that reported from other countries, it nevertheless raises concerns, given the sizable evidence of the short-term impact of heavy cannabis use on memory and learning, and its negative role on academic and health outcomes of university students [19,68]. 

Comparisons of findings between IDU studies are not straight forward. The literature reveals many groupings of the illicit substance/s that are used. For instance, some studies separate marijuana or cannabis use from each other IDU [65,69]. Others refer to a mesh of terms e.g., neuroenhancing substances (could include coffee, energy drinks, nicotine, alcohol) or psycostimulants [70]; illicit stimulants (amphetamines, Fenethylline, Ritalin, Concerta, Dexedrine, Adderall, Vyvanse) [71]; nonmedical attention deficit hyperactivity disorder (ADHD) medication [72]; illicit substances (including cocaine, designer drugs, and nonmedical use of prescription stimulants and opioids) [73]; illicit drug use, and misuse of prescription stimulants, sedatives, and opioids [74]; hallucinogen use [75]; smart drugs (aka cognitive enhancers, prescription drugs taken, either without a prescription or at a dose exceeding that which is prescribed) [76,77]; street drugs vs. prescription drugs [78]; or nonmedical use of prescription stimulants [79]. 

In addition to the nature of the illicit substance used, comparisons are further compounded by the type/manner of use e.g., illicit use vs. medical use [71], as well as the various definitions of frequency of use: e.g., experimental, episodic, frequent [80]; or regular, occasional, and never use [28]. A related point is the changes in the legal context of marijuana use and recreational marijuana legalization (RML) [74] in some countries/states, and whether marijuana would be included in IDU or otherwise, particularly that the prevalence of marijuana use increased more after legalization among college students in RML vs. non-RML states [81,82]. 

A final issue is the pattern of reporting of the use of alcohol and illicit drug/s, where studies report the simultaneous use of the two substances together [83], or report IDU and tobacco use e.g., cannabis-only, concurrent cannabis and cigarette, simultaneous cannabis and cigarette users, and non-recent users of either substance [84]. While such reporting of co-use is useful as substances are usually not used in isolation, the additional reporting of individual substances alone aids the accurate comparisons across studies. Future agreements about the classification of substances, manner of use, and patterns of use as well as these other issues will assist more precise comparisons across different studies and time.

As for socio-demographic variables, we observed that males were 1.82 time more likely to report ever IDU than females. This finding supports the sex differences in marijuana use among college students, where about twice more men than women use it daily [5]. Evidence shows that marijuana use is often higher among male college students compared to females [85,86], although some studies did not find differences in marijuana use by sex among college students [3]. Our findings are consistent with previous research; illicit substance users among students at 8 German universities were predominately male [1]. A literature review found that males tend to consume significantly more amounts of all kinds of drugs, with the exception of tranquilizers [87]. Likewise, khat chewing was practiced more by males compared to females [adjusted odds ratio (AOR) 2.45, 95% CI 1.06–5.66)] [9]. Males have also been associated with khat chewing in other studies [88,89,90,91,92].

Among the current sample of young Finnish adults, students not living with parents during university semesters were significantly more likely to report ever IDU (AOR 2.59, P < 0.001). These observations agree with other research among college students, where living with one’s parents was shown to be a protective factor regarding illicit substance use among college students [10,93]. In Brazil, others similarly found that students who do not use psychoactive drugs were more likely to live with their parents [87], and familial support was protective for prescription-type opioids use among university students [94]. Living with one’s parents was shown to be protective factors concerning ISU among college students [10,11,93]. Living with parents is usually associated with tighter parental supervision, and higher levels of support and guidance to their young adult children during their college years, factors that might act as deterrents to IDU.

We found that students reporting lower religiosity were significantly more likely to report ever IDU (AOR 1.49, P = 0.022), supporting others, where religiosity was protective for illicit substance use among college students in Brazil and Iran [11,95]. Students who do not use psychoactive drugs are more likely to practice religious beliefs [87]. Similar evidence across seven universities in the United Kingdom suggests that religiosity was a protective factor against high alcohol consumption among students [50]. Likewise, others reported that religiosity was negatively correlated with the number of substances used in the past month [69]. 

We observed that higher level of religiosity was more common among those who reported never IDU (41.8%) compared to regular IDU (31.3%) (data not presented), in agreement with research in Berlin, where college students’ self-reported level of religiosity was significantly higher in non-users of illicit substance/s than in all other user groups and was the lowest in regular-users [69]. Social and instrumental support offered by faith and places of worship may help people abstain from substance use [96], and religiosity were among the factors perceived by youth to prevent substance use or help them quit using substances [97]. The mechanism by which religiosity bestows protection against substance use remains to be understood although others suggested that teaching of religious values and strong family structures associated with religiosity (providing social support and parental monitoring) may have protective impact in terms of substance use [98,99]. 

Surprisingly, we observed that those with higher health awareness were significantly more likely to report ever IDU (AOR 1.49, P = 0.022); in contrast to a study that used the same questionnaire, where those with higher health awareness were significantly less likely to report occasional IDU (AOR 0.71, P = 0.008) [28]. Likewise, our Finnish students who were single were significantly less likely to report ever IDU, again in contrast to other research that showed that married individuals were less likely to use drugs [100]. These two findings could be due to that our sample of ever IDU users actually comprised much fewer (17) regular users and much more (228) occasional users.

In terms of health variables, in the current study, students with high depressive symptoms were significantly more likely to report ever IDU (AOR 1.82, P = 0.004). We agree with others, where among university students in Brazil, IDU was positively associated with major depressive episode [101]; and having higher depressive symptoms score was associated with ever IDU among students in North Kosovo [102]. Others found that the presence of psychiatric disorders was positively associated with the extent of illicit substance use [93]. Among college students, depression is often associated with significant problems including substance use [103,104], and students with higher levels of depressive symptoms were more likely to use cannabis in an emotional pain or sex-seeking context [105]. Nevertheless, among second-year medical students in France, there was no correlation between depression and the consumption of cannabis [106].

As for lifestyle variables, across our sample, daily smoking was an independent significant predictor of ever IDU (AOR 3.69, P < 0.001). In terms of predictive factors for the presence and extent of illicit substance use, others similarly found that daily and occasional consumption of tobacco was highly associated with illicit substance use prevalence and number of currently consumed substances [69]. Being a smoker was associated with ever IDU [102], and our findings are consistent with other research that showed positive associations between tobacco use, and illicit substance use, suggesting that smoking tobacco is a marker for other risky and addictive behaviour [10,28,33].

In the current study, students reporting heavy episodic drinking during the last 2 weeks and those with possible alcohol dependence were significantly more than twice as likely to report ever IDU (AOR 2.38 and 2.55 respectively, P < 0.001 for both). In the UK, research across seven universities, using the same questionnaire as in the current study, reported that regular IDU was significantly more likely among students with heavy episodic drinking or possible alcohol dependency [28]. Our findings support others where there was positive associations between heavy-drinking behaviour and ISU [33,107]; alcohol consumption was an independent predictor of recreational drug use among medical and nursing students in Cameroon (OR 5.08; 95% CI 1.54–16.73; P = 0.008) [108]; frequency of alcohol use predicted illicit drug experience among college students in Turkey [109]; and alcohol use was associated with ever IDU [102].

Across the current sample, financial burden/s was not an independent predictor of ever IDU, despite that young adults with more spending money and higher family income were more likely to use marijuana [10,110], but this was not the case for other studies of college students [3]. Likewise, age was not an independent predictor of ever IDU in the current study, even though age had independent and consistent positive associations with occasional and regular IDU, while exhibiting a negative association with never IDU [28]. Similarly, perceived stress did not independently predict ever IDU in the present study, in partial agreement with other research where perceived stress were not associated with regular IDU but was significantly negatively associated with occasional IDU [28].

Likewise, among our sample, academic performance did not independently predict ever IDU, although better academic performance was significantly associated with regular IDU in the United Kingdom, raising questions of whether illicit drug/s may be used for performance enhancing [28]. Finally, we observed that discipline of study did not independently predict ever IDU, where evidence suggests that lifetime cannabis use is more frequent among arts and social science students [29,30,31,32].

The current study has limitations. Data was collected at one university; the sample remains a convenience sample, hence generalizations need to be cautious; and the cross-sectional nature of the survey precludes that causal relationships can be inferred. Self-reporting assessed IDU; no biochemical/clinical validations were conducted. Students may have self-selected to partake in the survey due to a favorable bias toward IDU, although this is not very likely as the study was a general health survey. It is difficult to evaluate whether the current study could suffer from over or underestimation of IDU, although there seems to be overestimation of peer substance use among the college-age young adults [35,111]. Our response rate of 27% was based on the number of returned questionnaires in relation to the total number of students enrolled at the university. In agreement with others [69], it is not easy to calculate a rigorous response rate, as we did not have access to the exact numbers of students who were reached by the mailing lists of the university (enrolled students who are not actively studying anymore, spam-filters, many students not using faculty mail services at all). Hence, our 27% calculated response rate could be a conservative assessment, and the actual response rate may be higher. Other variables would have been beneficial to assess e.g., fraternity or sorority membership, peer influences, harm perceptions, and negative consequences from IDU, including driving under influence, being physically injured, experiencing memory loss, sexual assault, or unprotected sex [112,113,114]. Surveys of students have been subject to similar limitations. 

Despite these limitations, the study has important strengths as to the best of our knowledge, no previous study seems to have investigated in detail the relationships the prevalence, and correlates of regular and occasional IDU and the associations between such use and a wide range of socio-demographic, academic, and health and lifestyle characteristics across Finnish university students attending at many different faculties.

## 5. Conclusions

This study has described a range of socio-demographic, academic, and health and lifestyle characteristics of the sample and the lifetime prevalence of IDU among Finnish university students. Using bivariate analysis, the study appraised these variables by regular, occasional, and never IDU status; and, employing multiple logistic regression analysis, assessed the independent predictors of ever (lifetime) IDU. Ever (lifetime) IDU use among this sample of college students was 20.5%, which is concerning when considering the possible impact of IDU on students. The compelling independent predictors of ever (lifetime) IDU included those not living with parents during semesters, those with lower levels of religiosity, daily smokers and those with heavy episodic drinking or possible alcohol dependency (AOR range 2.38–3.69, all significant). Education and prevention messages need to emphasize negative consequences as means to reinforce continued abstinence from IDU. Prevention strategies are required to reduce risk, and health promotion efforts could focus on beliefs and expectations about IDU and target student groups at risk for more efficient and successful efforts. 

## Figures and Tables

**Table 1:** Socio-demographics and lifetime illicit drug/s use of university students in Finland.

	Lifetime Illicit Drug/s Use			
	Yes, Regularly *N* (Valid %)	Yes, A Few Times *N* (Valid %)	Never *N* (Valid %)	Total *N* (Valid %)
	17 (1.5)	228 (19.6)	921 (79.0)	1166 (100)
Sex				
Female	7 (0.9)	142 (17.4)	669 (81.8)	823 (70.4)
Male	10 (2.9)	82 (24.0)	249 (73)	346 (29.6)
Age, years *N* (M±SD)	17 (24.5 ± 5.1)	228 (23.5 ± 4)	919 (22.8 ± 5.5)	1164 (23 ± 5.2)
Year of study				
1st year	5 (0.9)	93 (17.0)	449 (82.1)	553 (47.2)
2nd year	8 (2.3)	75 (21.8)	261 (75.9)	344 (29.4)
3rd year	4 (1.6)	49 (19.8)	194 (78.5)	251 (21.4)
≥4th year	0	11 (47.8)	12 (52.2)	23 (2.0)
Discipline of Study at University				
Education and Law	2 (1.1)	35 (18.8)	149 (80.1)	188 (16.4)
Economics	2 (1.5)	31 (22.8)	103 (75.7)	138 (12.0)
Medicine	4 (2.4)	30 (18.2)	131 (79.4)	168 (14.6)
Technology and Science	3 (0.9)	51 (15.7)	271 (83.4)	328 (28.5)
Humanities	4 (1.2)	76 (23.3)	246 (75.5)	327 (28.5)
Accommodation during semester				
With parents	5 (1.3)	61 (15.6)	325 (83.1)	394 (33.7)
Not with parents	12 (1.6)	165 (21.5)	592 (77)	776 (66.3)
Marital status				
Married or in relationship	9 (1.5)	131 (22.2)	449 (76.2)	593 (50.7)
Single	8 (1.4)	94 (16.5)	468 (82.1)	576 (49.3)
Religiosity (Importance of religion in life)				
Strongly or somewhat agree/neither agree nor disagree	5 (1.1)	71 (15.5)	382 (83.4)	464 (39.8)
Strongly disagree/somewhat disagree	11 (1.6)	156 (22.3)	531 (76.1)	702 (60.2)
Financial burden/s				
No burden	12 (1.2)	170 (17.7)	779 (81.1)	971 (83.0)
Strong burden	5 (2.5)	57 (28.8)	136 (68.7)	199 (17.0)
Self-rated general health				
Poor/Fair	5 (5.7)	33 (37.9)	49 (56.3)	87 (7.4)
Good/very good/excellent	12 (1.1)	194 (18.1)	866 (80.8)	1083 (92.6)
Health awareness				
Not at all/not much	4 (2.5)	27 (17.2)	126 (80.3)	159 (13.6)
To some extent/very much	13 (1.3)	201 (20.1)	786 (78.6)	1009 (86.4)
Depressive symptoms				
low (<5th quintile)	9 (1.0)	160 (17.2)	761 (81.8)	939 (79.8)
High (≥5th quintile)	8 (3.4)	67 (28.5)	160 (68.1)	237 (20.2)
Perceived stress				
Low (<median)	6 (1.3)	76 (16.6)	377 (82.1)	463 (39.4)
High (≥median)	11 (1.6)	151 (21.4)	544 (77.1)	713 (60.6)
Smoking				
(Never/occasional)	12 (1.1)	191 (17.6)	881 (81.3)	1094 (93.7)
Daily	5 (6.8)	36 (48.6)	33 (44.6)	(6.3)
Heavy episodic drinking last 2 weeks				
<1 time	3 (0.8)	40 (10.8)	326 (88.3)	372 (33.8)
≥1 time	14 (1.9)	179 (24.8)	529 (73.3)	729 (66.2)
CAGE score				
<2 positive responses	4 (0.5)	132 (16.3)	672 (83.2)	815 (71.2)
≥2 positive (possible problem drinking)	13 (4.0)	95 (29.1)	218 (66.9)	329 (28.8)
CAGE score				
(<3 positive responses)	11 (1.1)	188 (18.2)	836 (80.8	1044 (91.3)
≥3 positive (possible alcohol dependence)	6 (6.1)	39 (39.4)	54 (54.5)	100 (8.7)
Academic performance compared to peers				
Same, better or much better	13 (1.3)	190 (19.3)	781 (79.4)	992 (84.6)
Worse or much worse	4 (2.2)	38 (21.3)	136 (76.4)	180 (15.4)

**Table 2:** Illicit drug/s use by selected variables among university students in Finland (bivariate analysis).

Variable	Illicit Drug/s Use											
	Yes, Regularly (*n* = 17)				Yes, a Few times (*n* = 228)				Never (*n* = 921)			
	*N* (%)	OR	P	95% CI	*N* (%)	OR	P	95 %CI	*N* (%)	OR	P	95 %CI
Sex (Female)	7 (0.9)				142 (17.4)				669 (81.8)			
Male	10 (2.9)	3.84	**0.007**	1.45–10.19	82 (24.0)	1.55	**0.005**	1.14–2.11	249 (73)	0.60	**0.001**	0.45–0.81
												
Age (years)		1.04	0.21	0.98–1.11		1.02	0.10	0.99–1.05		0.97	0.06	0.95–1.00
												
Accommodation during semester (With parents)	5 (1.3)				61 (15.6)				325 (83.1)			
(Not with parents)	12 (1.6)	1.32	0.61	0.46–3.77	165 (21.5)	1.49	**0.02**	1.08–2.05	592 (77)	0.68	**0.02**	0.50–0.93
												
Marital status (Married/in relationship)	9 (1.5)				131 (22.2)				449 (76.2)			
Single	8 (1.4)	0.85	0.75	0.33–2.23	94 (16.5)	0.69	**0.01**	0.51–0.93	468 (82.1)	1.43	**0.01**	1.08–1.91
												
Financial burden/s (No burden)	12 (1.2)				170 (17.7)				779 (81.1)			
Strong burden	5 (2.5)	2.39	0.11	0.83–6.88	57 (28.8)	1.92	**<0.0001**	1.35–2.73	136 (68.7)	0.51	**<0.0001**	0.36–0.72
												
Religiosity (Strongly or somewhat agree/neither agree nor disagree)	5 (1.1)				71 (15.5)				382 (83.4)			
Strongly disagree/somewhat disagree	11 (1.6)	1.58	0.40	0.55–4.59	156 (22.3)	1.58	**0.004**	1.16–2.16	531 (76.1)	0.63	**0.003**	0.47–0.86
												
Discipline of Study at University (Humanities)	4 (1.2)				76 (23.3)				246 (75.5)			
Education and Law	2 (1.1)	0.83	0.83	0.15–4.56	35 (18.8)	0.76	0.23	0.49–1.19	149 (80.1)	1.31	0.23	0.84–2.03
Economics	2 (1.5)	1.19	0.84	0.22–6.62	31 (22.8)	0.97	0.91	0.61–1.57	103 (75.7)	1.02	0.95	0.64–1.62
Medicine	4 (2.4)	1.88	0.38	0.46–7.63	30 (18.2)	0.74	0.22	0.46–1.19	131 (79.4)	1.25	0.33	0.80–1.97
Technology and Science	3 (0.9)	0.68	0.62	0.15–3.07	51 (15.7)	0.61	**0.01**	0.41–0.90	271 (83.4)	1.63	**0.01**	1.11–2.40
												
Self-rated general health (Poor/Fair)	5 (5.7)				33 (37.9)				49 (56.3)			
Good/very good/excellent	13 (1.3)	0.14	**<0.0001**	0.05–0.40	194 (18.1)	0.33	**<0.0001**	0.21–0.53	866 (80.8)	3.26	**<0.0001**	2.08–5.11
												
Health awareness (Not at all/not much)	4 (2.5)				27 (17.2)				126 (80.3)			
To some extent/very much	13 (1.3)	0.52	0.26	0.17–1.63	201 (20.1)	1.19	0.44	0.77–1.86	786 (78.6)	0.90	0.64	0.59–1.38
												
Smoking (Never/occasional)	12 (1.1)				191 (17.6)				881 (81.3)			
Daily	5 (6.8)	11.13	**<0.0001**	3.70–33.41	36 (48.6)	5.03	**<0.0001**	3.06–8.28	33 (44.6)	0.19	**<0.0001**	0.11–0.30
												
Depressive symptoms (low < 5th quintile)	9 (1.0)				160 (17.2)				761 (81.8)			
High (≥5th quintile)	8 (3.4)	4.23	**0.003**	1.61–11.13	67 (28.5)	1.99	**<0.0001**	1.43–2.78	160 (68.1)	0.47	**<0.0001**	0.34–0.65
												
Perceived stress Low (<median)	6 (1.3)								377 (82.1)			
High (≥median)	11 (1.6)	1.27	0.64	0.47–3.47		1.38	**0.04**	1.02–1.87	544 (77.1)	0.73	**0.04**	0.54–0.98
												
Academic performance compared to peers (Same, better or much better)	13 (1.3)				190 (19.3)				781 (79.4)			
Worse or much worse	4 (2.2)	1.77	0.33	0.57–5.50	38 (21.3)	1.15	0.49	0.78–1.70	136 (76.4)	0.84	0.37	0.58–1.23
												
Heavy episodic drinking last 2 weeks (<1 time)	3 (0.8)				40 (10.8)				326 (88.3)			
≥1 time	14 (1.9)	2.88	0.10	0.82–10.08	179 (24.8)	2.76	**<0.0001**	1.91–3.99	529 (73.3)	0.36	**<0.0001**	0.25–0.52
												
CAGE score (<2 positive responses)	4 (0.5)				132 (16.3)				672 (83.2)			
≥2 positive (possible problem drinking)	13 (4.0)	10.02	**<0.0001**	3.23–31.05	95 (29.1)	2.22	**<0.0001**	1.64–3.01	218 (66.9)	0.41	**<0.0001**	0.30–0.55
												
CAGE score (<3 positive responses)	11 (1.1)				188 (18.2)				836 (80.8			
≥3 positive (possible alcohol dependence)	6 (6.1)	8.45	**<0.0001**	3.01–23.70	39 (39.4)	3.21	**<0.0001**	2.07–4.99	54 (54.5)	0.29	**<0.0001**	0.19–0.44

OR: odds ratio; CI confidence interval; bolded cells indicate statistical significance.

**Table 3:** Predictors of ever (lifetime) illicit drug/s use among university students in Finland *.

Variable	Ever Illicit Drug/s Use		
	AOR	95% CI for AOR	P
Sex (Female)	1		
Male	1.82	1.28–2.59	0.001
Accommodation during semester (With parents)	1		
(Not with parents)	2.59	1.72–3.92	<0.001
Marital status (Married/in relationship)	1		
Single	0.51	0.35–0.74	<0.001
Religiosity (Strongly or somewhat agree/neither agree nor disagree)	1		
Strongly disagree/somewhat disagree	1.49	1.06–2.09	0.022
Self-rated general health (Poor/Fair)	1		
Good/very good/excellent	0.41	0.23–0.74	0.003
Health awareness (Not at all/not much)	1		
To some extent/very much	1.93	1.14–3.27	0.014
Smoking (Never/occasional)	1		
Daily	3.69	2.10–6.48	<0.001
Depressive symptoms (low < 5th quintile)	1		
High (≥5th quintile)	1.82	1.21–2.75	0.004
Heavy episodic drinking last 2 weeks (<1 time)	1		
≥1 time	2.38	1.59–3.56	<0.001
CAGE score (<3 positive responses)	1		
≥3 positive (possible alcohol dependence)	2.55	1.57–4.15	<0.001

* Multiple logistic regression identified significant factors associated with ever (lifetime) IDU. OR for ever IDU was adjusted for all variables in the tables (sex, age, discipline of study at university, accommodation during-semester, marital status, religiosity status, financial burden/s, self-rated general health, health awareness, depressive symptoms, perceived stress, smoking, heavy episodic drinking in the last 2 weeks, CAGE score, and academic performance compared to one’s peers); AOR=Adjusted odds ratio; CI=Confidence Interval; Hosmer & Lemshow Chi-Square = 13.4; P = 0.1 (indicating adequate fit of the model to the data).

## References

[1] Schilling L., Zeeb H., Pischke C., Helmer S., Schmidt-Pokrzywniak A., Reintjes R., Walter U., Girbig M., Krämer A., Icks A. (2017). Licit and illicit substance use patterns among university students in Germany using cluster analysis. Subst. Abus. Treat. Prev. Policy.

[2] Locke G.W., Shilkret R., Everett J.E., Petry N.M. (2015). Interpersonal guilt and substance use in college students. Subst. Abus..

[3] Phillips K.T., Lalonde T.L., Phillips M.M., Schneider M.M. (2017). Marijuana use and associated motives in Colorado University students. Am. J. Addict..

[4] Pickard M., Bates L., Dorian M., Greig H., Saint D. (2000). Alcohol and drug use in second-year medical students at the University of Leeds. Med. Educ..

[5] Schulenberg J.E., Johnston L.D., O’Malley P.M., Bachman J.G., Miech R.A., Patrick M.E. Monitoring the Future National Survey Results on Drug Use, 1975–2016: Volume II, College Students and Adults Ages 19–55.

[6] Pearson M.R., Liese B.S., Dvorak R.D. (2017). Marijuana Outcomes Study Team. College student marijuana involvement: Perceptions, use, and consequences across 11 college campuses. Addict. Behav..

[7] Caldeira K.M., Arria A.M., O’Grady K.E., Vincent K.B., Wish E.D. (2008). The occurrence of cannabis use disorders and other cannabis-related problems among first-year college students. Addict. Behav..

[8] Vorster A., Gerber A.M., van der Merwe L.J., van Zyl S. (2019). Second and third year medical students’ self-reported alcohol and substance use, smoking habits and academic performance at a South African medical school. Health SA.

[9] Adere A., Yimer N.B., Kumsa H., Liben M.L. (2017). Determinants of psychoactive substances use among woldia university students in Northeastern Ethiopia. BMC Res. Notes.

[10] Suerken C.K., Reboussin B.A., Sutfin E.L., Wagoner K.G., Spangler J., Wolfson M. (2014). Prevalence of marijuana use at college entry and risk factors for initiation during freshman year. Addict. Behav..

[11] Mohammadpoorasl A., Ghahramanloo A.A., Allahverdipour H., Augner C. (2014). Substance abuse in relation to religiosity and familial support in Iranian college students. Asian J. Psychiatr..

[12] Sommet A., Ferrieères N., Jaoul V., Cadieux L., Soulat J.M., Lapeyre-Mestre M., Montastruc J.L. (2012). Use of drugs, tobacco, alcohol and illicit substances in a French student population. Therapie.

[13] Bajwa H.Z., Al-Turki A.S., Dawas A.M., Behbehani M.Q., Al-Mutairi A.M., Al-Mahmoud S., Shukkur M., Thalib L. (2013). Prevalence and factors associated with the use of illicit substances among male university students in Kuwait. Med. Princ. Pract..

[14] Maier L.J., Liechti M.E., Herzig F., Schaub M.P. (2013). To dope or not to dope: Neuroenhancement with prescription drugs and drugs of abuse among Swiss university students. PLoS ONE.

[15] Gupta S., Sarpal S.S., Kumar D., Kaur T., Arora S. (2013). Prevalence, pattern and familial effects of substance use among the male college students—A north Indian study. J. Clin. Diagn. Res..

[16] Center for Behavioral Health Statistics and Quality (2015). 2014 National Survey on Drug Use and Health: Detailed Tables.

[17] National Institute on Drug Abuse n.d., Drug Facts. What is Marijuana?. https://www.drugabuse.gov/publications/drugfacts/marijuana.

[18] World Health Organization (WHO) (2004). Neuroscience of Psychoactive Substance Use and Dependence Summary.

[19] Arria A.M., Caldeira K.M., Bugbee B.A., Vincent K.B., O’Grady K.E. (2015). The academic consequences of marijuana use during college. Psychol. Addict. Behav..

[20] Arria A.M., Garnier-Dykstra L.M., Calderia K.M., Vincent K.B., Winick E.R., O’Grady K.E. (2013). Drug use patterns and continuous enrollment in college: Results from a longitudinal study. J. Stud. Alcohol Drugs.

[21] Arria A.M., Wilcox H.C., Calderia K.M., Vincent K.B., Garnier-Dykstra L.M., O’Grady K.E. (2013). Dispelling the myth of “smart drugs”: Cannabis and alcohol use problems predict nonmedical use of prescription stimulants for studying. Addict. Behav..

[22] Bell R., Wechsler H., Johnston L.D. (1997). Correlates of college student marijuana use: Results of a US national survey. Addiction.

[23] Phillips K.T., Phillips M.M., Lalonde T.L., Tormohlen K.N. (2015). Marijuana use, craving, and academic motivation and performance among college students: An in-the- moment study. Addict. Behav..

[24] Buckner J.D., Ecker A.H., Cohen A.S. (2010). Mental health problems and interest in marijuana treatment among marijuana-using college students. Addict. Behav..

[25] Wechsler H., Lee J.E., Nelson T.F., Kuo M. (2002). Underage college students’ drinking behavior, access to alcohol, and the influence of deterrence policies. Findings from the Harvard School of Public Health College alcohol study. J. Am. Coll. Health.

[26] Hingson R.W., Zha W., Weitzman E.R. (2009). Magnitude of and trends in alcohol- related mortality and morbidity among U.S. College students ages 18–24, 1998–2005. J. Stud. Alcohol Drugs Suppl..

[27] Substance Abuse and Mental Health Services Administration, Center for Behavioral Health Statistics and Quality (2013). National Estimates of Drug-Related Emergency Department Visits, 2004–2011–Illicits (Excluding Alcohol).

[28] El Ansari W., Vallentin-Holbech L., Stock C. (2015). Predictors of Illicit Drug/S Use among University Students in Northern Ireland, Wales and England. Glob. J. Health Sci..

[29] Nelson T., Wechsler H. (2001). Alcohol and college athletes. Med. Sci. Sport Exerc..

[30] Newbury-Birch D., Walshaw D., Kamali F. (2001). Drink and drugs: From medical students to doctors. Drug Alcohol Depend..

[31] Yusko D., Buckman J., White H., Pandina R. (2008). Alcohol, tobacco, illicit drugs, and performance enhancers: A comparison of use by college student athletes and nonatheletes. J. Am. Coll. Health.

[32] Webb E., Ashton H., Kelly P., Kamali F. (1997). Patterns of alcohol consumption, smoking and illicit drug use in British university students: Interfaculty comparisons. Drug Alcohol Depend..

[33] Primack B.A., Kim K.H., Shensa A., Sidani J.E., Barnett T.E., Switzer G.E. (2012). Tobacco, marijuana, and alcohol use in university students: A cluster analysis. J. Am. Coll. Health.

[34] Varga M.D. (2012). Adderall abuse on college campuses: A comprehensive literature review. J. Evid. Based Soc. Work.

[35] American College Health Association (2018). National College Health Assessment II: Fall 2017 Reference Group Executive Summary.

[36] Gebremariam T.B., Mruts K.B., Neway T.K. (2018). Substance use and associated factors among Debre Berhan University students, Central Ethiopia. Subst. Abus. Treat. Prev. Policy.

[37] El Ansari W., Sebena R., Labeeb S. (2014). Multiple risk factors: Prevalence and correlates of alcohol, tobacco and other drug (ATOD) use among university students in Egypt. J. Subst. Use.

[38] El Ansari W., Salam A., Suominen S. (2020). Is alcohol consumption associated with poor perceived academic performance? Survey of undergraduates in Finland. Int. J. Environ. Res. Public Health.

[39] Nyström M., Peräsalo J., Salaspuro M. (1994). Mixed use of psychiatric drugs and alcohol by finnish university students participating in a health screening. Scand. J. Prim. Health Care.

[40] Kopteff P.J. (1980). A survey of the abuse of medicines and illicit drugs by Finnish students. Int. J. Addict..

[41] European Monitoring Centre for Drugs and Drug Addiction (EMCDDA) Country Overview: Finland. Country Overview for Finland: Situation Summary. http://www.emcdda.europa.eu/publications/country-overviews/fi#drd.

[42] Academy of Finland Research Programme on Substance Use and Addictions, 2006. http://www.aka.fi/Tiedostot/Tiedostot/ADDIKTIO/Ohjelmamuistio%20%28pdf%29.pdf..

[43] Onyeka I.N., Beynon C.M., Hannila M.L., Tiihonen J., Föhr J., Tuomola P., Kuikanmäki O., Tasa N., Paasolainen M., Kauhanen J. (2014). Patterns and 14-year trends in mortality among illicit drug users in Finland: The HUUTI study. Int. J. Drug Policy.

[44] El Ansari W., Stock C., Snelgrove S., Hu X., Parke S., Davies S., John J., Adetunji H., Stoate M., Deeny P. (2011). Feeling healthy? A survey of physical and psychological wellbeing of students from seven universities in the UK. Int. J. Environ. Res. Public Health.

[45] El Ansari W., Labeeb S., Kotb S., Yousafzai M.T., El-Houfey A., Stock C. (2012). Correlates of smoking, quit attempts and attitudes towards total smoking bans at university: Findings from eleven faculties in Egypt. Asian Pac. J. Cancer Prev..

[46] El Ansari W., Stock C. (2012). Factors associated with smoking, quit attempts and attitudes towards total smoking bans at university: A survey of seven universities in England, Wales and Northern Ireland. Asian Pac. J. Cancer Prev..

[47] El Ansari W., Sebena R., Stock C. (2013). Socio-demographic correlates of six indicators ofalcohol consumption: Survey findings of students across seven universities in England, Wales and Northern Ireland. Arch. Public Health.

[48] El Ansari W., Stock C., Mills C. (2013). Is alcohol consumption associated with poor academic achievement in university students?. Int. J. Prev. Med..

[49] El Ansari W., Dibba E., Labeeb S., Stock C. (2014). Body image concern and its correlates among male and female undergraduate students at assuit university in Egypt. Glob. J. Health Sci..

[50] El Ansari W., Sebena R., Stock C. (2014). Do importance of religious faith and healthy lifestyle modify the relationships between depressive symptoms and four indicators of alcohol consumption? A survey of students across seven universities in England, Wales, and Northern Ireland. Subst. Use Misuse.

[51] El Ansari W., Berg-Beckhoff G. (2019). Association of health status and health behaviors with weight satisfaction vs. body image concern: Analysis of 5888 undergraduates in Egypt, Palestine, and Finland. Nutrients.

[52] El Ansari W., Samara A. (2018). Adherence to recommended dietary guidelines and the relationships with the importance of eating healthy in Egyptian University students. Int. J. Prev. Med..

[53] El Ansari W., Stock C. (2010). Is the health and wellbeing of university students associated with their academic performance? Cross sectional findings from the United Kingdom. Int. J. Environ. Res. Public Health.

[54] El Ansari W., Ssewanyana D., Stock C. (2018). Behavioral health risk profiles of undergraduate university students in England, Wales, and Northern Ireland: A cluster analysis. Front. Public Health.

[55] Mikolajczyk R.T., Maxwell A.E., Naydenova V., Meier S., El Ansari W. (2008). Depressive symptoms and perceived burdens related to being a student: Survey in three European Countries. Clin. Pract. Epidemiol. Ment. Health.

[56] American College Health Association (2007). American College Health Association National College Health Assessment (ACHA-NCHA): Spring 2006 reference group report (abridged). J. Am. Coll. Health.

[57] Stock C., Kücük N., Miseviciene I., Guillén-Grima F., Petkeviciene J., Aguinaga-Ontoso I., Krämer A. (2003). Differences in health complaints between University students from three European countries. Prev. Med..

[58] Beck A.T., Steer R.A., Ball R., Ranieri W. (1996). Comparison of beck depression inventories—IA and II in psychiatric outpatients. J. Pers. Assess..

[59] Schmitt M., Beckmann M., Dusi D., Maes J., Schiller A., Schonauer K. (2003). (Messgu’te des vereinfachten Beck- Depressions-Inventars (BDI-V). Diagnostica.

[60] Cohen S., Kamarck T., Mermelstein R. (1983). A global measure of perceived stress. J. Health Soc. Behav..

[61] Hurrelmann K., Kolip P. (1994). Der Jugendgesundheitssurvey. Presseinformationsdienst Des SFB 227.

[62] Winther Ringgard L., Birk Nissen S., Nielsen G.A. (2005). Unges Livsstil Og Dagligdag 2003.

[63] Ewing J.A. (1984). Detecting alcoholism. The CAGE questionnaire. JAMA.

[64] National Survey on Drug Use and Health 2016. Detailed Tables. www.samhsa.gov/data/sites/default/files/NSDUH-DetTabs-2016/NSDUH-DetTabs-2016.htm.

[65] Kwan M.Y.W., Faulkner G.E.J., Arbour-Nicitopoulos K.P., Cairney J. (2013). Prevalence of health-risk behaviours among Canadian post-secondary students: Descriptive results from the National College Health Assessment. BMC Public Health.

[66] National Institute on Drug Abuse (2010). Monitoring the Future 2009.

[67] Bennasar-Veny M., Yañez A.M., Pericas J., Ballester L., Fernandez-Dominguez J.C., Pedro Tauler P., Aguilo A. (2020). Cluster analysis of health-related lifestyles in university students. Int. J. Environ. Res. Public Health.

[68] Arria A.M., Caldeira K.M., Bugbee B.A., Vincent K.B., O’Grady K.E. (2016). Marijuana use trajectories during college predict health outcomes nine years post- matriculation. Drug Alcohol Depend..

[69] Viohl L., Ernst F., Gabrysch J., Petzold M.B., Köhler S., Ströhle A., Betzler F. (2019). ‘Higher education’—Substance use among Berlin college students. Eur. J. Neurosci..

[70] Kusturica J., Hajdarević A., Nikšić H., Skopljak A., Tafi Z., Kulo A. (2019). neuroenhancing substances use, exam anxiety and academic performance in Bosnian-Herzegovinian first-year university students. Acta Med. Acad..

[71] Alrakaf F.A., Binyousef F.H., Altammami A.F., Alharbi A.A., Shadid A., Alrahili N. (2020). Illicit Stimulant Use among Medical Students in Riyadh, Saudi Arabia. Cureus.

[72] Rabiner D.L., Anastopoulos A.D., Costello E.J., Hoyle R.H., McCabe S.E., Swartzwelder H.S. (2009). Motives and perceived consequences of nonmedical ADHD medication use by college students: Are students treating themselves for attention problems?. J. Atten. Disord..

[73] Kollath-Cattano C., Hatteberg S.J., Kooper A. (2020). Illicit drug use among college students: The role of social norms and risk perceptions. Addict. Behav..

[74] Alley Z.M., Kerr D.C.R., Bae H. (2020). Trends in college students’ alcohol, nicotine, prescription opioid and other drug use after recreational marijuana legalization: 2008–2018. Addict. Behav..

[75] Grant J.E., Lust K., Chamberlain S.R. (2019). Hallucinogen use is associated with mental health and addictive problems and impulsivity in university students. Addict. Behav. Rep..

[76] Hildt E. (2013). Cognitive Enhancement–A Critical Look at the Recent Debate, Cognitive Enhancement.

[77] Champagne J., Gardner B., Dommett E.J. (2019). Modelling predictors of UK undergraduates’ attitudes towards smart drugs. Trends Neurosci. Educ..

[78] Rosansky J.A., Rosenberg H. (2019). University students’ self-reported reasons for abstinence from prescription and non-prescription stimulants and depressants. Drug Alcohol Depend..

[79] Benson K., Flory K., Humphreys K.L., Lee S.S. (2015). Misuse of stimulant medi- cation among college students: A comprehensive review and meta-analysis. Clin. Child Fam. Psychol. Rev..

[80] Gerra G., Benedetti E., Resce G., Potente R., Cutilli A., Molinaro S. (2020). Socioeconomic status, parental education, school connectedness and individual socio-cultural resources in vulnerability for drug use among students. Int. J. Environ. Res. Public Health..

[81] Kerr D.C.R., Bae H., Koval A.L. (2018). Oregon recreational marijuana legalization: Changes in undergraduates’ marijuana use rates from 2008 to 2016. Psychol. Addict. Behav..

[82] Miller A.M., Rosenman R., Cowan B.W. (2017). Recreational marijuana legalization and college student use: Early evidence. SSM Popul. Health.

[83] Colomer-Pérez N., Chover-Sierra E., Navarro-Martínez R., Andriusevičienė V., Vlachou E., Cauli O. (2019). Alcohol and drug use in European University health science students: Relationship with self-care ability. Int. J. Environ. Res. Public Health.

[84] Ruglass L.M., Espinosa A., Fitzpatrick S., Meyer M.K., Cadet K., Sokolovsky A., Jackson K.M., White H.R. (2020). Prevalence and correlates of concurrent and simultaneous cannabis and cigarette use among past-year cannabis-using us college students. Subst. Use Misuse.

[85] Keith D.R., Hart C.L., McNeil M.P., Silver R., Goodwin R.D. (2015). Frequent marijuana use, binge drinking and mental health problems among undergraduates. Am. J. Addict..

[86] McCabe S.E., Morales M., Cranford J.A., Delva J., McPherson M.D., Boyd C.J. (2007). Race/ethnicity and gender differences in drug use and abuse among college students. J. Ethn. Subst. Abus..

[87] Candido F.J., Souza R., Stumpf M.A., Fernandes L.G., Veiga R., Santin M., Kluthcovsky A. (2018). The use of drugs and medical students: A literature review. Rev. Assoc. Med. Bras..

[88] Haile D., Lakew Y. (2015). Khat chewing practice and associated factors among adults in Ethiopia: Further analysis using the 2011 demographic and health survey. PLoS ONE.

[89] Damena T., Mossie A., Tesfaye M. (2011). Khat chewing and mental distress: A community-based study, in Jimma city, southwestern Ethiopia. Ethiop. J. Health Sci..

[90] Gebreslassie M., Feleke A., Melese T. (2013). Psychoactive substances use and associated factors among Axum university students, Axum Town North Ethiopia. BMC Public Health.

[91] Gebrehanna E., Berhane Y., Worku A. (2014). Khat chewing among Ethiopian University Students—A growing concern. BMC Public Health.

[92] Lakew A., Tariku B., Deyessa N., Reta Y. (2014). Prevalence of Catha edulis (Khat) chewing and its associated factors among ataye secondary school students in northern Shoa, Ethiopia. Adv. Appl. Sociol..

[93] Kendler K.S., Edwards A., Myers J., Cho S.B., Adkins A., Dick D. (2015). The predictive power of family history measures of alcohol and drug problems and internalizing disorders in a college population. Am. J. Med. Genet..

[94] Abbasi-Ghahramanloo A., Khodadost M., Moradpour F., Karimirad M.R., Kamali R., Ziarati F. (2018). Prevalence of nonmedical use of prescription-type opioids, methylphenidate, and sedative-hypnotics among university students in the south of Iran: A regression analysis. Electron. Physician.

[95] Gomes F.C., Andrade A.G.D., Izbicki R., Almeida A.M., Oliveira L.G.D. (2013). Religion as a protective factor against drug use among Brazilian University students: A national survey. Rev. Bras. Psiquiatr..

[96] Mak H.W. (2019). Dimensions of religiosity: The effects of attendance at religious services and religious faith on discontinuity in substance use. J. Stud. Alcohol Drugs.

[97] Loffredo C.A., Boulos D.N., Saleh D.A., Jillson I.A., Garas M., Loza N., Samuel P., Shaker Y.E., Ostrowski M.J., Amr S. (2015). Substance use by Egyptian youth: Current patterns and potential avenues for prevention. Subst. Use Misuse.

[98] Walker C., Ainette M.G., Wills T.A., Mendoza D. (2007). Religiosity and substance use: Test of an indirect-effect model in early and middle adolescence. Psychol. Addict. Behav..

[99] Geppert C., Bogenschutz Michael P., Miller William R. (2009). Development of a bibliography on religion, spirituality and addictions. Drug Alcohol Rev..

[100] Martin C.C. (2019). High socioeconomic status predicts substance use and alcohol consumption in U.S. undergraduates. Subst. Use Misuse.

[101] Flesch B.D., Houvèssou G.M., Munhoz T.N., Fassa A.G. (2020). Major depressive episode among university students in Southern Brazil. Rev. Saude Publica.

[102] Gazibara T., Milic M., Parlic M., Stevanovic J., Lazic D., Maric G., Kisic-Tepavcevic D., Pekmezovic T. (2018). Illict drug use and academia in North Kosovo: Prevalence, patterns, predictors and health-related quality of life. PLoS ONE.

[103] Cranford J.A., Eisenberg D., Serras A.M. (2009). Substance use behaviors, mental health problems, and use of mental health services in a probability sample of college students. Addict. Behav..

[104] Martins S.S., Fenton M.C., Keyes K.M., Blanco C., Zhu H., Storr C.L. (2012). Mood and anxiety disorders and their association with non-medical prescription opioid use and prescription opioid-use disorder: Longitudinal evidence from the national epidemiologic study on alcohol and related conditions. Psychol. Med..

[105] Beck K.H., Caldeira K.M., Vincent K.B., O’Grady K.E., Wish E.D., Arria A.M. (2009). The social context of cannabis use: Relationship to cannabis use disorders and depressive symptoms among college students. Addict. Behav..

[106] Vaysse B., Gignon M., Zerkly S., Ganry O. (2014). Alcohol, tobacco, cannabis, anxiety and depression among second-year medical students. Identify in order to act. Sante Publique.

[107] Grossbard J.R., Mastroleo N.R., Kilmer J.R., Lee C.M., Turrisi R., Larimer M.E., Ray A. (2010). Substance use patterns among first-year college students: Secondary effects of a combined alcohol intervention. J. Subst. Abus. Treat..

[108] Mbanga C.M., Efie D.T., Aroke D., Njim T. (2018). Prevalence and predictors of recreational drug use among medical and nursing students in Cameroon: A cross sectional analysis. BMC Res. Notes.

[109] Ayvasik H.B., Sümer H.C. (2010). Individual differences as predictors of illicit drug use among Turkish college students. J. Psychol..

[110] Ramo D.E., Delucchi K.L., Hall S.M., Liu H., Prochaska J.J. (2013). Marijuana and tobacco co-use in young adults: Patterns and thoughts about use. J. Stud. Alcohol Drugs.

[111] Sanders A., Stogner J.M., Miller B.L. (2013). Perception vs. reality: An investigation of the misperceptions concerning the extent of peer novel drug use. J. Drug Educ..

[112] Welsh J.W., Shentu Y., Sarvey D.B. (2019). Substance use among college students. Focus Am. Psychiatr. Publ..

[113] LaBrie J.W., Kenney S.R., Mirza T., Lac A. (2011). Identifying factors that increase the likelihood of driving after drinking among college students. Accid. Anal. Prev..

[114] Soule E.K., Barnett T.E., Moorhouse M.D. (2015). Protective behavioral strategies and negative alcohol-related consequences among US college fraternity and sorority members. J. Subst. Use.

